# A79 EAT, SLEEP, WORK, REPEAT! WEARABLES AND APPS TO TRACK IBD PATIENTS’ SYMPTOMS, DIET, SLEEP, AND PHYSICAL ACTIVITY

**DOI:** 10.1093/jcag/gwae059.079

**Published:** 2025-02-10

**Authors:** P M Miranda, I Fernando, D Armstrong

**Affiliations:** Medicine, McMaster University, Hamilton, ON, Canada; Medicine, McMaster University, Hamilton, ON, Canada; Medicine, McMaster University, Hamilton, ON, Canada

## Abstract

**Background:**

Lifestyle factors like diet, physical activity, and sleep patterns are known to influence symptoms and progression in Inflammatory Bowel Disease (IBD). However, accurately determining temporal relationships between these factors and symptoms has been hampered by a lack of reliable data collection methods. New technologies, such as wearables and smartphone apps, offer real-time, remote monitoring and allow for better understanding of their relationship with IBD symptoms.

**Aims:**

To assess the feasibility of using remote monitoring tools (wearables and mobile apps) to explore the temporal relationships between symptoms, diet, and sleep in IBD patients.

**Methods:**

Adult IBD patients participated in a 3-month pilot study (Track-IBD), during which they wore a fitness monitor (Oura Ring) and tracked their diet and symptoms in real-time using Keenoa (diet tracking) and Zamplo (symptom tracking) smartphone apps.

**Results:**

Fifteen IBD patients (9 - Crohn’s disease, 6 - ulcerative colitis; 8 female; 12 Caucasian; mean age 41.2 years) were enrolled. The usage rates were 69.8% for Zamplo, 77.0% for Keenoa, and 77.0% for the Oura Ring. The most commonly reported symptoms were abdominal pain, diarrhea, and fatigue, which together accounted for 67% of all recorded symptoms. Symptoms were predominantly reported in the afternoon (30.1%) and late-night (49.2%), while morning and early-night periods saw fewer reports (20.7%). Most symptoms occurred within 5 hours of a meal, with peaks in meal-to-symptom latency (MTSL) at 0.5–1 hour and 4–5 hours post-meal, demonstrating an association between meal timing and symptoms. No correlation was found between MTSL and the nature of the symptoms (upper, lower, or extra-intestinal) or macronutrient intake. Wearable data analysis revealed decreased sleep quality and increased average heart rate on symptomatic days (p<0.04). Most participants found the apps easy to use (Oura: 100%, Zamplo: 93%, Keenoa: 73%) and helpful in managing their IBD (Oura: 100%, Zamplo: 87%, Keenoa: 60%).

**Conclusions:**

The Track-IBD study demonstrates high adherence to monitoring diet, symptoms, and physiological measures by IBD patients over 3 months. It also shows the feasibility of combining multiple tracking technologies to assess lifestyle patterns, physiological parameters, and symptoms in real-time. Preliminary analyses identified relationships between symptoms, diet and sleep quality, highlighting the potential to identify lifestyle triggers of disease and symptom flares and provide personalized therapeutic advice to IBD patients.

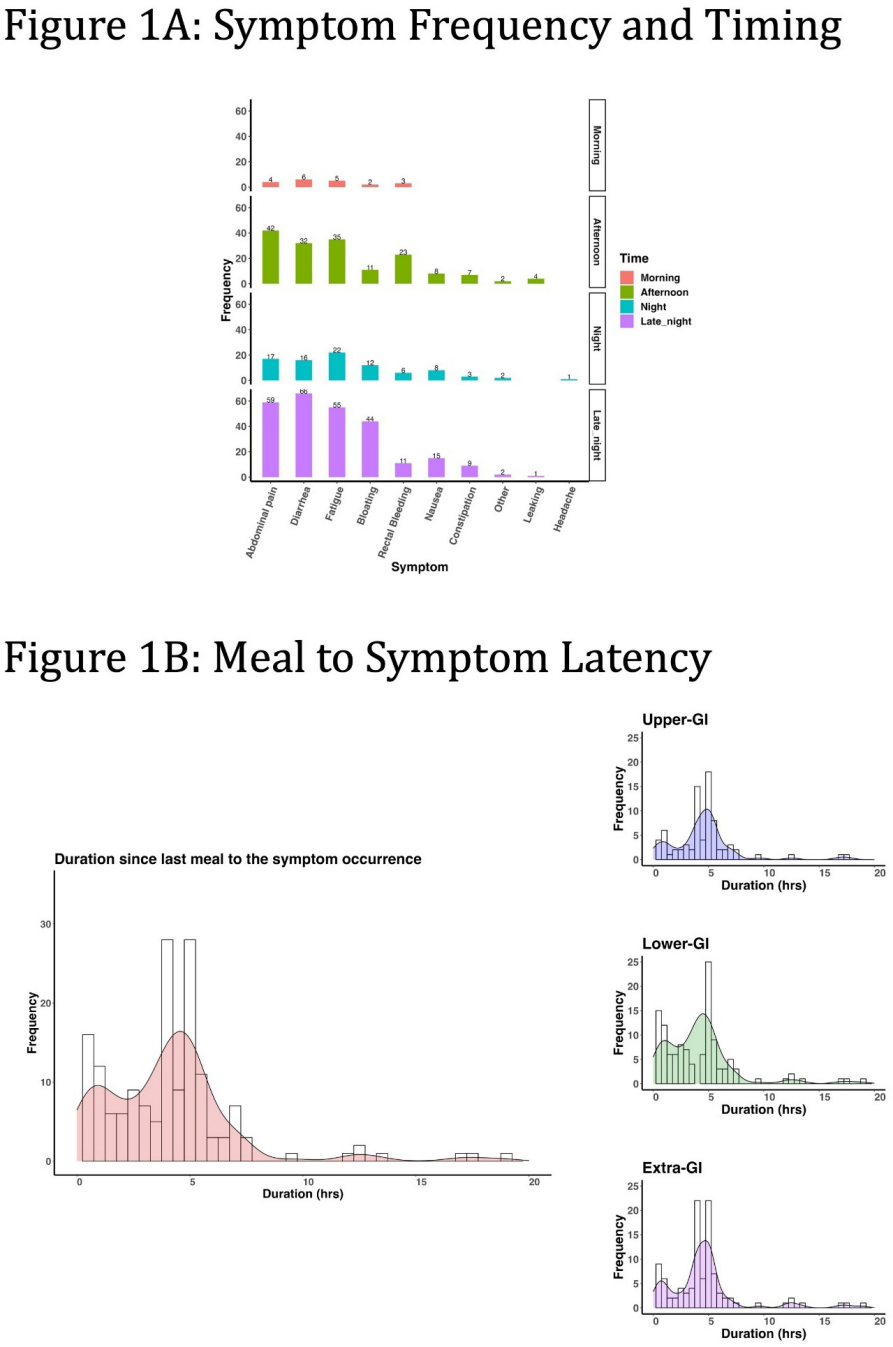

**Funding Agencies:**

Balsam Foundation

